# Medical Lexicon or Modern Terminology; Being a Complete Vocabulary of Definitions, Including All the Technical Terms Employed by Writers and Teachers of Medical Science at the Present Day, and Comprising Several Hundreds of Words Not Found in Any Other Dictionary

**Published:** 1845-02

**Authors:** 


*Medical Lexicon or Modern Terminology; being a complete Vocabulary of Definitions, including all the technical terms employed by Writers and Teachers of Medical Science at the present day, and comprising several hundreds of words not found in any other Dictionary. Designed for the use of Students and Practitioners.* By David Meredith Reese, A. M., M. D., of New York. Late Professor of the Institutes of Medicine and Surgery and Medical Jurisprudence in the Washington University of Baltimore, Editor of Cooper's Surgical Dictionary, &c. 24mo. pp. 229. New York, 1845.

We do not think we have ever seen so unsuccessful an attempt at authorship, with so presumptuous a title, as in the case before us. Admitting, that a vocabulary of terms in medicine might be needed—which we think disputable—a production like this is totally unfitted for supplying the deficiency; and this, not alone on account of its diminutive size, but of its positive errors. "To diminish the number of ambiguous and unpronounceable technicals"—says the Author—"would, indeed, be a desideratum; and yet the pedantry and false pretension of medical exclusives, who still abound in the profession, would frown upon any such effort to popularize the science. Still, however, it may be lawful to attempt the exclusion of obsolete though" [?] "ancient terms, multitudes of which are not found in any modern standard author, nor employed by any public lecturer in the United States. In the present work, this latter attempt has been made, and with what success the profession will judge.

"For the more ancient terminological nomenclatures and obsolete technicalities, the student will find occasion for reference to the larger works of Motherby, Parr, Fox, Morris, Quincy, Hooper, Coxe, Dunglison, Hoblyn, &c. But he will seldom be disappointed in this little manual, should he search for any term retained by modern authors, or employed by his teachers during his collegiate course."

This is strong self-commendation; and yet the appeal which the Author makes to the Profession cannot be responded to in the same tone. It is impracticable for us to do more than select a comparatively few examples, which will amply indicate whether he be competent for the difficult task which he has assumed. And FIRST, as regards the omission of obsolete words. What shall we say of the retention of such terms as the following? *Abracadabra, Accessus, Acestor, Acestoris, Æger, Ægrotus, Ægra, Ægrota, Afflatus, Agrippæ, Album balsamum, Album græcum, Album nigrum, Altheus, Anosia, Apella, &c. &c.*, all in a portion of the terms under the letter A.

Again: why should French terms, like *Abaissement de la matrice, Abouchement, &c. &c.* be retained, whilst others, so much more employed, are discarded.

Another fault is the glaring insufficiency of the definitions in multitudes of cases, which can convey no adequate idea of the meaning of the term. To take a few examples:—

ABDUCENS OCULI; "A muscle of the Eye."

ABDUCENS LABIORUM; "A muscle of the Lips."

ABDUCTOR INDICIS MANUS; "Muscle of the Fingers."

AFFINITY; "chemical attraction."

The serious, fatal, objection to the book, however, is its numerous, flagrant, and unpardonable errors; for which we can find no palliation for one who has assumed to be a lexicographer. We doubt, indeed, whether a single page could be pointed out which does not contain one or more. Take page 13 at random, which comprises the words between Acus, 'needle,' and Adipsia, 'absence of thirst,' and we find the following:

"Acute; "a rapid and severe disease, a sharp and pungent pain, an active form of inflammation." That is, the word acute, instead of being an adjective is a noun, signifying all these things!

"Additamentum; "superadded, as the prolongation of certain sutures of the skull." That is, Additamentum, instead of signifying 'an addition,' means 'superadded.' The substantive here takes the place of the adjective!

"Adeps Suillæ" for Adeps Suilla.

"Adeps Ovilli" for Adeps Ovilla.

"Adeps preparata" for Adeps præparata.

"Adipose; "fatty, the cellular tissue and its contents." That is, 'Adipose' means "the cellular tissue and its contents!" And these are taken from one page only!

It would be impracticable to specify the multitude of errors comprised in this small—infinitesimally small—production. We may, however, enumerate a few more to those already instanced; for example: Ædema for *Œdema;* Ær, 'the atmosphere, air;' Æriform, "applied to gases;" Æthmoid for Ethmoid; Ague, chill, the cold stage of an intermittent; *Aiquille* for *Aiguille;* 'a needle;' Alvo solvens; "the bowels being confined;" Anœmia; "bloodlessness;" Anœsthesia for Anæsthesia; Aneurism cordis for Aneurisma cordis; Aneurism verum; Aneurism spurium; Aneurism varicosum; Anhœmia for Anhæmia; Asthma spasmodica; Balneum demibain; 'hip-bath;' *Bruit de Placentaire;* "utero-placental murmur in auscultation;" Cacæthes for Cacoëthes; Catarrh senilis; Chordæ tendinæ; Corda Tympani; "nerve of the ear, vidian branch of the portio dura [!];" Corpus pampiniform; Cystirrhæa; Diebus llternus; "every other day;" Diebus tertius; "every third day, &c.;" Diosphyros Virginiana; Dosis maximum; Dosis minimum:—but it is idle to proceed farther. And then how rich and expressive to the young student such definitions as the following, to say nothing of the accuracy of some of them. Bulbous

ROOT; 'used medicinally;' CUBITUM IRE; "to go to bed;" CULINARY; "appertaining to the kitchen;" DESMOID TISSUE; "ligamentous, aponeurotic;" DIABETES; "morbid urinary secretion, containing oxalic acid;" [!] EUSTACHIAN TUBE; "canal leading from the throat to the internal [!] ear;" TOXICODENDRON; "sumach, poisonous rhus;" TRACHEAL; "structures connected with the windpipe;"—*et sic passim.*

Enough has been adduced to show that the Author evidently possesses no qualifications for the task; no knowledge of what a Dictionary ought to be; and that he is grievously ignorant of the most common classical words. The work is, indeed, utterly beneath contempt, and we caution our readers accordingly.

